# Separation of Lignin from *Paulownia* and Its Application in DES Gels

**DOI:** 10.3390/gels12050365

**Published:** 2026-04-27

**Authors:** Hanyin Li, Liangdi Zhang, Xiaobo Xue, Yi Meng, Youming Dong, Fei Xiao, Hanmin Wang, Cheng Li

**Affiliations:** 1 College of Forestry, Henan Agricultural University, Zhengzhou 450002, China; 2 College of Materials Science and Engineering, Nanjing Forestry University, Nanjing 210037, China; 3 Hunan Academy of Forestry, Changsha 410018, China; 4 State Key Laboratory of Bio-Based Fiber Materials, Tianjin Key Laboratory of Pulp and Paper, Tianjin University of Science and Technology, Tianjin 300457, China

**Keywords:** deep eutectic solvent, pretreatment, lignin, DES gel

## Abstract

In this study, binary and ternary DES systems were prepared using choline chloride (ChCl) with lactic acid (LA), glycerol (GL), urea, and acrylic acid (AA) to extract lignin from Paulownia. The chemical structure of lignin was analyzed to evaluate the structural changes induced by various DES systems, and the isolated lignin was used to prepare DES gels. The results showed that lignin extracted using different DES systems shares similarities in its basic structural framework, with all samples retaining an intact benzene ring structure. However, there are certain differences in the content of the linking bonds and the S/G ratio, and the acidic DES caused the breakage of the β-O-4′ linkage in the lignin molecule, promoting its separation. The molecular weight distribution varied among the DES systems. In the ternary DES, the addition of acrylic acid disrupted lignin’s internal chemical linkages, leading to the precipitation of relatively small lignin molecules. TGA results demonstrated varying levels of thermal resistance among lignin extracted from different DES systems varied, with the best stability observed for lignin extracted from the ChCl-LA system. Lignin extracted from *Paulownia* using different DES systems was added to the DES gels, and the effects of lignin structure on the properties of the DES gels were investigated. The mechanical, swelling, microstructural, and thermal properties of DES gels prepared from different *Paulownia* lignin structures showed slight differences; however, no significant discrepancies were observed among the gels. The present work offers a novel strategy for the valorization of lignin derived from lignocellulosic biomass.

## 1. Introduction

Lignin, as the major component of lignocellulosic biomass, is the predominant naturally occurring aromatic macromolecule on earth. It possesses a highly complex three-dimensional network structure and diverse functional groups, offering numerous advantages such as biodegradability, non-toxicity, and biocompatibility [1]. Lignin also exhibits high stability, antioxidant properties, and antibacterial activity, making it highly promising for applications in agriculture, pharmaceuticals, and environmental engineering [2]. *Paulownia* is a fast-growing hardwood species widely distributed around the world. In Europe, it is mostly known and cultivated as an ornamental tree because of its rapid growth, attractive appearance and beautiful flowers [3]. However, its potential as a high-quality lignin resource has long been underestimated. Recently, an increasing number of studies have recognized *Paulownia* as a promising lignocellulosic feedstock for biorefinery applications due to its rapid growth, high biomass yield, and favorable chemical composition [4]. Compared with other common lignin sources such as agricultural residues, softwoods and conventional hardwoods, *Paulownia* shows obvious advantages: it has a fast growth rate, high biomass yield, abundant resources, and does not compete with food crops for cultivated land. In addition, the lignin in *Paulownia* has a suitable content and a relatively complete aromatic skeleton structure, which is beneficial to the efficient extraction and subsequent high-value utilization. Therefore, using *Paulownia* biomass as a raw material for lignin extraction can not only expand the application field of this underutilized tree species, but also provide a new renewable resource for the development of green biomass refining. The efficient separation of lignin is a crucial step in its valorization; however, due to its complex structure and the covalent and hydrogen-bonding interactions between lignin molecules and other biomass main components, lignin is highly challenging to isolate from other constituents. Traditional lignin separation methods still have limitations, such as harsh reaction conditions, equipment degradation, and suboptimal extraction efficiency. Additionally, during the extraction process, condensation reactions often occur, leading to reduced yields and purity and significantly hindering the high-value utilization of lignin [5].

Lately, deep eutectic solvents (DES) as the new solvent systems have gained attracting increasing attention from researchers for their green, low-cost, and biodegradable properties, along with their physicochemical characteristics that are similar to those of traditional ionic liquids. These solvents are formulated by combining a hydrogen bond acceptor (HBA) with a hydrogen bond donor (HBD) [6,7]. Compared to traditional ionic liquids, DESs offer several advantages: the components used to form DES solutions are generally non-toxic and cost-effective, and their mixing induces a substantial melting-point depression, thereby enhancing the safety of experimental operations [8]. Choline chloride (ChCl) is an economical, efficient, safe, and biodegradable quaternary ammonium compound that frequently functions as a hydrogen bond acceptor (HBA) during the preparation of DES. Unlike other components, ChCl can be extracted from biomass and mixed with substances that act as HBDs, such as urea, carboxylic acids, and alcohols, to form ChCl-based DES, which have been extensively applied in biomass pretreatment processes [9]. Extensive studies demonstrate that applying DES to pretreat lignocellulosic biomass effectively disrupt ether bonds between biomass components, selectively dissolve lignin, and leave carbohydrates largely unaffected, resulting in the effective recovery of high-purity lignin while preserving the integrity of cellulose [10]. These characteristics make DES an ideal solvent for biomass pretreatment and demonstrate its great potential in lignin extraction. However, due to the varying methods of lignin separation, lignin from different sources exhibits significant differences in chemical composition, structure, and properties [11,12]. Given the complexity and structural heterogeneity of lignin, its utilization often faces numerous challenges that limit its industrial applications. Therefore, in-depth knowledge regarding lignin structure is indispensable to efficiently convert and valorize it.

Lignin is an abundant natural resource, yet the majority of it is still burned directly as fuel in industrial production, resulting in significant waste [13]. This practice has prompted a growing interest in the high-value utilization of lignin, emerging as a primary direction in current lignin research [14]. Due to its excellent biocompatibility, antibacterial properties, antioxidant activity, and high stability, lignin offers numerous advantages. Additionally, the phenylpropane structural units in lignin are predominantly connected by carbon-carbon and ether bonds, and they contain functional groups including carboxyl and phenolic hydroxyl, which possess strong metal-chelating abilities [15,16]. These structural features make lignin highly advantageous for ion adsorption when incorporated into hydrogels, thereby enhancing the mechanical strength of the hydrogels. Consequently, lignin has demonstrated considerable potential in improving the performance of hydrogels [17,18,19]. Deep eutectic solvent (DES) gels are composite gels that combine the properties of low eutectic solvents and hydrogels, resulting from the compatibility and interaction between DES and hydrogels [20]. Thanks to the inherent physicochemical properties of DES, DES gels exhibit superior environmental stability compared to traditional hydrogels, along with advantages such as good thermal stability, conductivity, and repeatable deformability [21]. Recent studies have progressively begun exploring the application of lignin in DES gels. For example, Yan et al. [20] used a ternary DES (consisting of choline chloride, glycerol, and Lewis acid) for lignocellulose pretreatment. The resulting lignin-enriched DES extract then functioned as the reaction medium to fabricate a polyacrylic acid–polyvinyl alcohol (PAA-PVA) double-network gel, which exhibited outstanding mechanical robustness, self-adhesiveness, and high electrochemical sensitivity. Their study found that the incorporation of DES mixture conferred the gel with superior electro-sensing capabilities and antifreeze properties, allowing it to maintain excellent pliability and efficient transport even at −20 °C, with a high gauge factor of 4.19, making it suitable for flexible sensors. Huang et al. [22] used polyacrylamide (PAM) as a cross-linking network in a DES-based solution containing lithium salts to prepare a new polymer electrolyte DES gel. Their research revealed that the addition of lignin significantly enhanced the efficacy of DES gel-based electrolyte devices, with the prepared devices exhibiting a wide voltage window (0 to 2.4 V) and a maximum energy density of 42.6 Wh/kg. This research provides new insights regarding the development of sustainable, high-energy-density flexible devices. Additionally, Wang et al. [23] used a DES composed of betaine and ethylene glycol to dissolve lignin. The polymerization of acrylic acid (AA) was catalyzed by the dynamic redox reaction between Fe_2_(SO_4_)_3_ and lignin in the DES system at room temperature. The resulting composite lignin-DES gel showed significant improvement in conductivity, with conductivity increasing from 0.09 ms/cm to 4.07 ms/cm over the temperature range of −20 °C to 120 °C. Furthermore, the gel exhibited a remarkable tensile strength reaching approximately 1.8 MPa, coupled with an exceptional strain capability exceeding 1000%. These gels, with good conductivity, high strength, and thermal stability, show great promise for use in supercapacitors and wearable sensors. Thus, lignin has demonstrated substantial potential in enhancing the performance of DES gels.

This study addresses the low efficiency of lignin component separation and the significant resource waste in lignocellulosic biomass. It uses different deep eutectic solvents (DES) systems to isolate lignin from *Paulownia* and its subsequent incorporation into DES gels. The structure of the extracted lignin is characterized, and the effects of varying lignin structures on the performance of the DES gels are evaluated. The research primarily involves preparing various DES systems to pretreat *Paulownia* biomass, isolate and extract lignin, and investigate the structural characteristics of lignin using two-dimensional nuclear magnetic resonance (2D NMR), gel permeation chromatography (GPC), Fourier-transform infrared spectroscopy (FTIR), and thermogravimetric analysis (TGA) to examine the impact of different DES pretreatments on lignin structure. Lignin extracted from different DES systems is then incorporated into DES gels. Choline chloride acts as a hydrogen bond acceptor, while acrylic acid serves both as a polymer monomer and a hydrogen bond donor. These components form a DES solution through hydrogen bonding. N,N′-Methylenebisacrylamide (MBA) is used as a cross-linker, and ammonium persulfate (APS) as an initiator to prepare gels incorporated with lignin. The mechanical properties, microstructure, chemical structure, and thermal stability of the DES gels are tested and analyzed to explore how different lignin structures affect their performance. This study broadens the scope of the advanced valorization of lignin resources and establishes a theoretical basis for the comprehensive development and efficient utilization of all components of *Paulownia* biomass.

## 2. Results and Discussion

### 2.1. Lignin Isolation from Paulownia Wood Using Different DES Systems and Structural Analysis

#### 2.1.1. Fourier Transform Infrared Spectroscopy Analysis of Lignin

**Figure 1:**
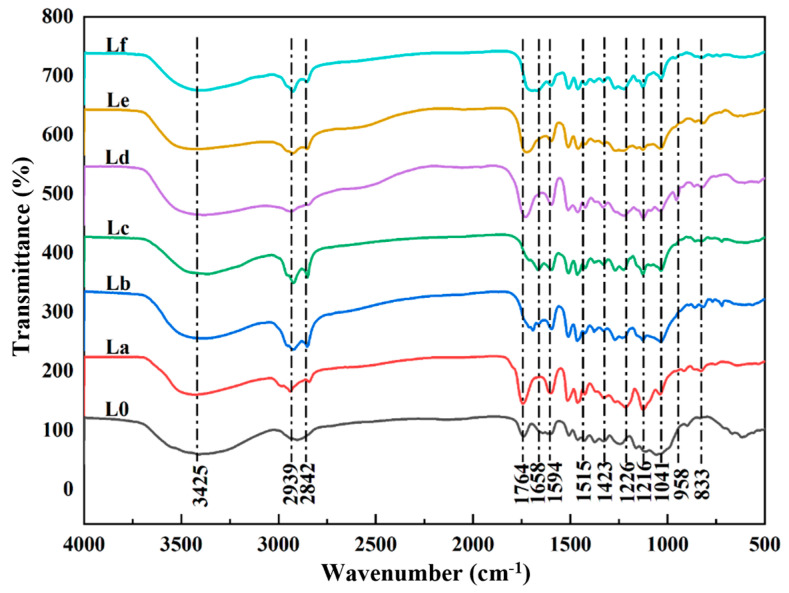
Infrared spectra of different lignins and *Paulownia* raw materials.

Figure 1 shows the FT-IR spectra of the *Paulownia* lignin samples extracted after DES treatment and the raw *Paulownia* material. The absorption peaks at 1594, 1515, and 1423 cm^−1^ are characteristic peaks for the aromatic ring skeletal vibrations of lignin. The peak at 3425 cm^−1^ originates from the stretching vibrations of aliphatic and phenolic -OH groups. The peaks at 2939 and 2842 cm^−1^ are assigned to the stretching behavior of C-H bonds within methyl and methylene structures, respectively [24]. The peak at 1764 cm^−1^ is due to the C=O stretching vibration of non-conjugated ketones, carbonyl, and ester groups in the lignin structure. It can be seen that the absorption peaks for the Lb and Lc samples are almost absent or have disappeared at this band, whereas those for La, Ld, Le and Lf are enhanced, which may be due to esterification reactions occurring between the acidic DES and the lignin side chains, resulting in the formation of additional C=O bonds [25]. The peaks at 1594, 1515, and 1423 cm^−1^ are attributed to the benzene ring structure in lignin, indicating that it remains intact after DES treatment [26]. The peak at 1216 cm^−1^ results from the stretching vibrations of C-C, C-O, and C=O bonds, and the peaks at 958 and 833 cm^−1^ are due to out-of-plane deformational modes of the aromatic C-H bonds [27]. The results demonstrate that the spectral positions and their typical signals of the lignin samples extracted after different DES treatments are quite similar, suggesting that the lignin extracted after DES treatment retains a relatively intact benzene ring structure.

#### 2.1.2. Molecular Weight Analysis and Yield of Lignin

As shown in Table 1, the weight-average molecular weight (Mw) and number-average molecular weight (Mn) of lignins extracted by different DES systems vary. Among them, the lignin La has the highest Mw. According to the 2D NMR results (Table 2), the ChCl-LA DES system caused the rupture of a large number of β-O-4′ linkage bonds in lignin, leading to the depolymerization of La lignin. Its molecular structure also shows a higher number of β-5′ linkage bonds, indicating that condensation reactions occurred, forming additional β-5′ linkages. This observation suggests that during the ChCl-LA pretreatment process, both depolymerization and condensation reactions of lignin occurred simultaneously, with the latter dominant, resulting in a higher molecular weight of La lignin. The Mn and Mw of lignin extracted by the binary DES system are higher than those from the ternary DES system, possibly because the addition of acrylic acid in the ternary system led to more β-O-4′ linkage cleavage in lignin. As a result, lignin macromolecules underwent depolymerization and precipitated as relatively lower-molecular-weight compounds [28]. The polydispersity index (PDI) of lignin is closely related to the molecular weight distribution of lignin. A smaller PDI indicates a more uniform and concentrated molecular weight distribution, whereas a larger PDI suggests a broader distribution [29]. In the binary DES system, the PDI of lignin extracted from ChCl-GL (Lb) is 2.60, which is the highest among the six lignins, indicating that the lignin fragments extracted by the ChCl-GL DES system are of uneven size. On the other hand, the PDI of lignin extracted from ChCl-LA-AA in the ternary DES system (Ld) is the lowest at 1.35, suggesting that the lignin extracted by the ChCl-LA-AA DES system has a more uniform and concentrated molecular weight distribution. The significant differences in lignin extraction yield are mainly attributed to the distinct chemical properties of different DES systems. La and Ld samples are extracted by ChCl-LA and ChCl-LA-AA respectively, and both the DES systems contain lactic acid, a strong hydrogen-bond donor with carboxylic acid functionality. These acidic DES systems effectively cleave lignin-carbohydrate complexes (LCC) and promote the depolymerization and dissolution of lignin [30], resulting in high lignin yields (75.57% and 45.55%, respectively). The lower yield of Ld compared to La may be due to the substantial reduction in the overall acidity of the system. In contrast, other DES systems (close to neutral or basic DES) exhibit milder fractionation effects, resulting in relatively lower extraction efficiency. These findings are consistent with the literature, which reports that DES acidity and hydrogen-bond donor type strongly influence delignification efficiency [31,32].

#### 2.1.3. Two-Dimensional Nuclear Magnetic Resonance (2D NMR) Analysis of Lignin

**Figure 2:**
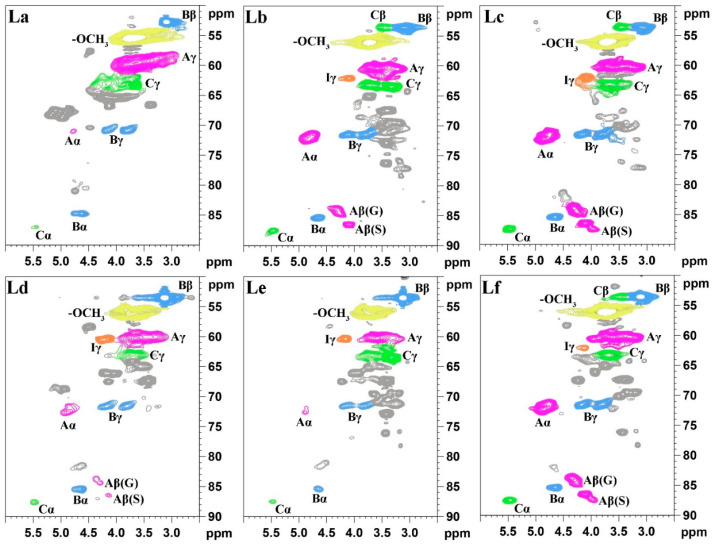
2D NMR spectra of different lignins (side chain regions, and the colors are correspond to the colors of the structure in Figure 4).

**Figure 3:**
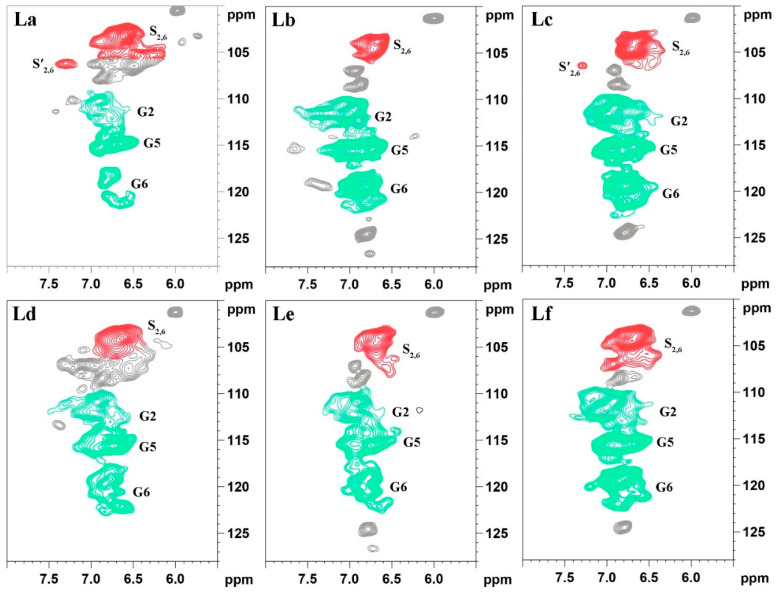
2D NMR spectra of different lignins (aromatic ring regions, and the colors are correspond to the colors of the structure in Figure 4).

**Figure 4:**
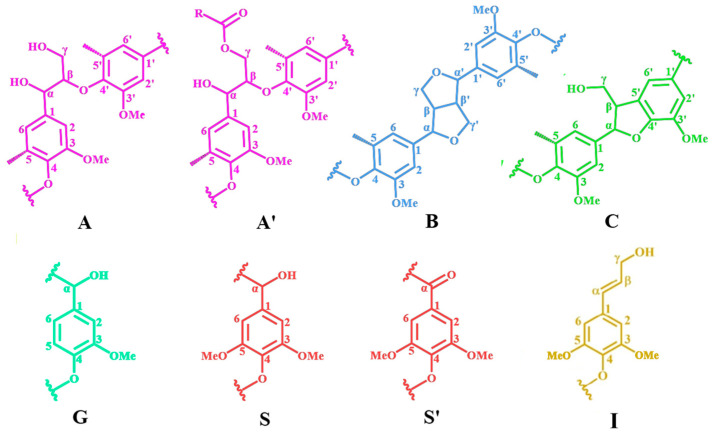
Major structural units in lignin samples.

Due to the high signal resolution of 2D-HSQC NMR, it can clearly depict the complex molecular structure of lignin through its spectral signals, making it a powerful tool for lignin characterization. In this experiment, 2D-HSQC NMR was used to analyze six different DES-extracted *Paulownia* lignin samples and examine differences in their molecular structures. Figure 2 and Figure 3 show the 2D NMR spectra of the side-chain region (*δ*_C_/*δ*_H_ 50.0–90.0/2.50–6.00 ppm) and the aromatic ring region (*δ*_C_/*δ*_H_ 100.0–128.0/5.50–8.00 ppm) for the six lignins, respectively. Figure 4 illustrates the main structural units in the lignin samples. The linkage types among the lignin structural units were mainly reflected in the side-chain spectral region, while the basic lignin structural units: syringyl (S) and guaiacyl (G) units were primarily ascribed to the aromatic ring region [33].

In the side-chain spectral region of the lignin molecules, signals related to the methoxy group (–OCH_3_, *δ*_C_/*δ*_H_ 55.6/3.71) and the β-O-4′ structure (aryl ether bond structure A) from lignin were observed. Additionally, the C–H signal related to the α position of β-O-4′ appeared at *δ*_C_/*δ*_H_ 71.9/4.81, and the γ position C–H signal appeared at *δ*_C_/*δ*_H_ 59.5–62.4/3.24–3.58. A C–H correlation signal corresponding to the γ position of the p-hydroxycinnamyl alcohol structure (I) was also detected at *δ*_C_/*δ*_H_ 61.5/4.10. For the β-β′ resinol structure, the α-position C–H signal peak was observed at *δ*_C_/*δ*_H_ 85.4/4.67 ppm, and the β-position C–H signal peak appeared at *δ*_C_/*δ*_H_ 53.5/3.12 ppm. The β-position C–H signals at *δ*_C_/*δ*_H_ 84.3/4.29 and 86.4/4.11 correspond to the G and S units in the β-O-4′ structure, respectively. The lignins La, Ld, and Le, extracted after ChCl-LA, ChCl-LA-AA, and ChCl-GL-AA DES treatments, show weaker signals of Aα in their side-chain regions, suggesting that under the acidic DES pretreatment, a large number of β-O-4′ structures in the lignin molecules underwent significant cleavage.

In the aromatic ring region of the lignin molecules, the signal intensities of the S and G units are notably high, demonstrating that the isolated lignin mainly consists of S and G units. The signal at *δ*_C_/*δ*_H_ 103.4/6.62 ppm corresponds to the 2,6 positions on the aromatic framework of the S unit, while the signal at *δ*_C_/*δ*_H_ 106.2/7.32 ppm is associated with the 2,6 positions of the oxidized syringyl unit’s aromatic ring. The signals at *δ*_C_/*δ*_H_ 110.3/6.92, 114.8/6.69, and 119.5/6.83 ppm originate from the 2, 5, and 6 positions of the guaiacyl unit’s aromatic ring, respectively.

As shown in Table 2, a quantitative analysis of the linkages in different lignins was conducted using 2D NMR, which provided the S/G ratio and the content of various linkages between lignin units. The analysis revealed the occurrence of β-O-4′, β-β′, and β-5′ linkages in the lignin fractions isolated via all the DES systems. According to the 2D NMR quantification of La, it has the fewest β-O-4′ linkages and the most β-5′ linkages, suggesting that under acidic DES pretreatment, a substantial breakdown of the β-O-4′ ether bonds took place within lignin molecules, while condensation reactions likely resulted in the formation of numerous β-5′ linkages. Furthermore, the S/G ratio of La is the highest, which may be due to the selective degradation of G units and the condensation of G-type lignin, leading to an increase in the S/G ratio and a reduction in the G unit signal [34].

#### 2.1.4. Thermogravimetric Analysis of Lignin

**Figure 5:**
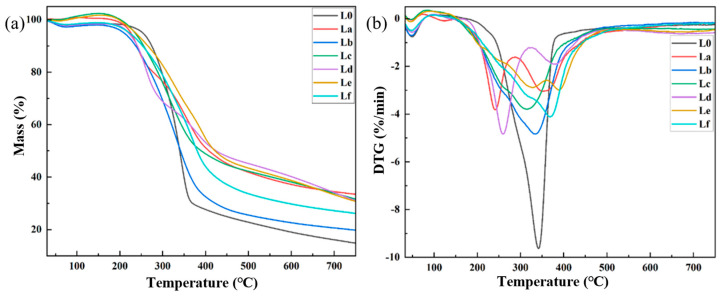
(**a**) TG curves of different lignins; (**b**) DTG curves of different lignins.

Figure 5 shows the thermogravimetric curves of the six different lignins. From the figure, it can be observed that lignins extracted with different DES systems exhibit similar overall trends during thermal degradation. The thermal pyrolysis of different lignins generally follows three stages: preheating, rapid pyrolysis, and residual carbon formation. First stage: From 30 to 200 °C the mass change of lignin is relatively stable, primarily attributed to the removal of both free and chemically bound water, accompanying with the release of volatile compounds within the lignin [35]. Second stage: From 200 to 500 °C there is a sharp weight reduction, which is largely attributable to the decomposition of phenolic, alcoholic, aldehyde, and carbohydrate components into CO, CO_2_, and CH_4_ [36]. Third stage: At temperatures above 500 °C this stage mainly represents the formation of residual carbon. The thermal weight loss is slower, with only a portion of the lignin undergoing pyrolysis. The remaining incombustible ash and solid coke remain, and their mass remains nearly unchanged [37].

The degradation process of the lignins extracted by different DES systems still follows the three-stage pattern of preheating, rapid pyrolysis, and residual carbon formation. However, the final ash content of the different lignins shows slight variation, with lignin La having the highest residual ash content, approximately 33%, and lignin Lb having the lowest, around 20%. Furthermore, the temperatures at which the maximum pyrolysis rate occurs for different lignins differ slightly, but overall, the maximum thermal degradation rate of lignin occurs around 300 °C These results indicate that the thermal stability of lignin extracted from *Paulownia* wood varies with the pretreatment DES system, with lignin extracted with the ChCl–LA DES system exhibiting the highest thermal stability.

### 2.2. Effect of Different Lignin Structures on DES Gels

#### 2.2.1. Mechanical and Swelling Properties of DES Gels

**Figure 6:**
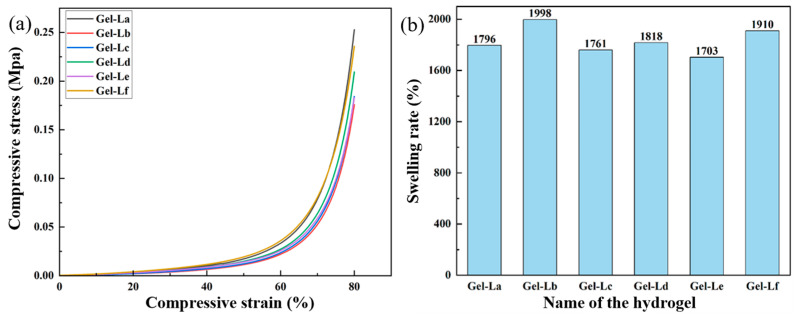
(**a**) Compression stress–strain diagram of DES gels with different kinds of lignin; (**b**) Swelling diagram of DES gels with different kinds of lignin.

The compressive stress–strain curves for DES gels incorporated with various lignins are depicted in Figure 6a. The data indicates that the strain increases with the increment of compressive stress. This behavior is categorizable into two phases: in the initial compression stage, as the load intensifies, the gel undergoes gradual contraction; when the compression strain exceeds 50%, the compression stress increases rapidly. This behavior is mainly related to the internal network of the DES gel and the hydrogen-bonding interactions between the DES and the polymer molecular chains [38]. The maximum compression stress of the six gels with different lignins ranges from 0.176 MPa to 0.252 MPa, with Gel-La exhibiting the highest. This result is primarily attributed to the increased molecular mass of lignin La. However, overall, the difference in maximum compression stress between the different gels is not significant, indicating that the lignin structure may not have a pronounced effect on the mechanical properties of the DES gels.

Water absorption swelling is a characteristic of gels, and the swelling ratio is a key indicator for evaluating them. The swelling ratio reveals the interactions between the macromolecular chains and different functional groups within the gel system [39,40]. The data in Figure 6b shows that the water absorption swelling ratios of the DES gels with different lignins added do not vary significantly, ranging between 1703% and 1998%. The reason for this minor difference is likely the relatively small amount of lignin added to the DES gels, which means the lignin structure does not have a noticeable effect on the gels’ water absorption and swelling performance.

#### 2.2.2. Microscopic Structure Analysis of DES Gels

**Figure 7:**
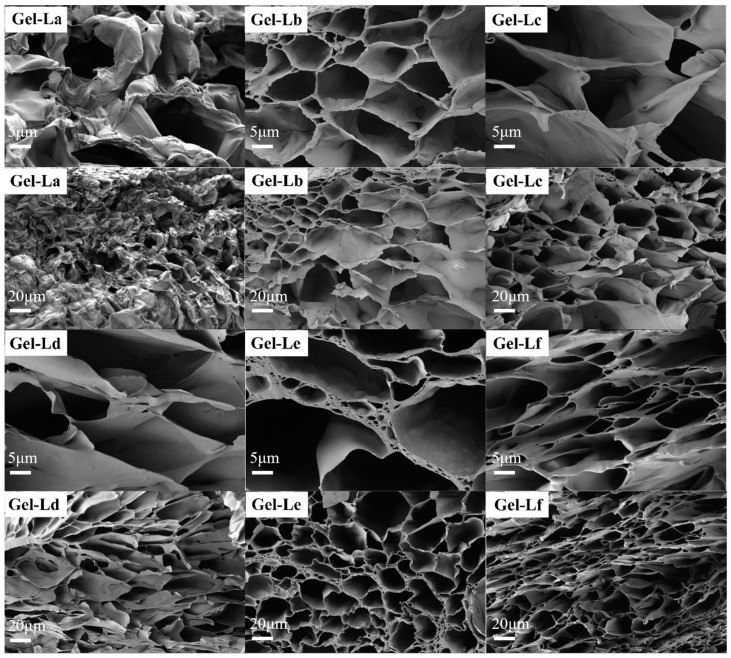
SEM of DES gels with different kinds of lignin.

The scanning electron micrographs (SEM) in Figure 7 illustrate the morphologies of DES gels with adding different lignin. Drying the gels under normal pressure results in significant shrinkage due to the collapse of internal pores as the gels lose water. This results in differences in the gel’s pore structure. By first freezing the DES gels at ultra-low temperatures and then using a freeze-dryer, water molecules can sublimate directly from the solid to the gas phase, allowing the DES gels to dry while preserving the integrity of their internal pore structure. As shown in the images, the freeze-dried DES gel sections still exhibit a honeycomb-like pore structure under SEM. However, the microscopic structures of the gels with different lignins added show some variation. This observation may be due to structural differences in lignin, with varying functional groups on lignin molecules influencing grafting and cross-linking interactions with acrylic acid. As a result, the internal pore structures of the DES gels differ.

#### 2.2.3. Infrared Spectroscopic Analysis of DES Gels

**Figure 8:**
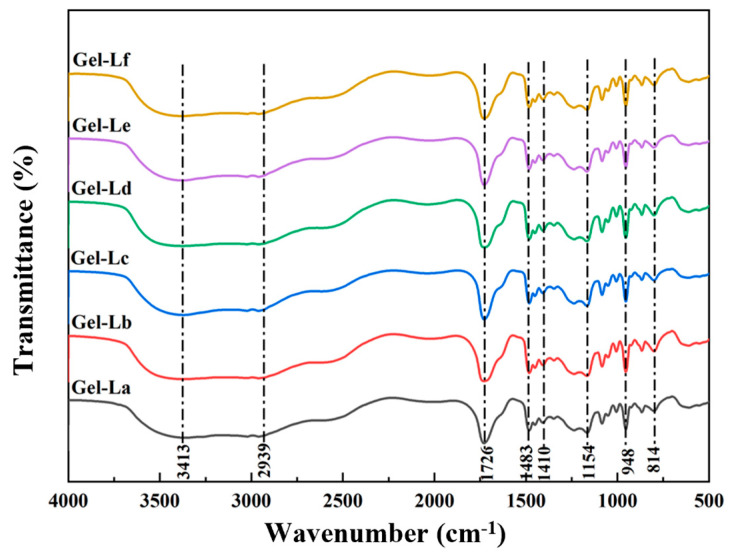
Infrared spectra of DES gels with different types of lignin.

Figure 8 shows the Fourier-transform infrared (FT-IR) spectra of DES gels containing lignin with different structures. The broad peak at 3413 cm^−1^ is primarily attributed to the stretching vibrations of –OH groups, which mainly originate from the hydrogen-bonding network in DES and may overlap with absorption peaks from the phenolic hydroxyl groups and aliphatic –OH groups in lignin [41]. The peak at 2939 cm^−1^ corresponds to C–H stretching vibrations, while the absorption band near 1726 cm^−1^ is attributed to carbonyl stretching vibrations. The absorption peak observed in the 1154 cm^−1^ region is related to C–O stretching vibrations [42]. The absorption bands near 1483 cm^−1^ and 1410 cm^−1^ are primarily associated with the bending vibrations of methyl/ethyl groups and the skeletal vibrations of the DES components themselves. Due to the relatively low lignin content, some of its characteristic absorption bands are likely masked by the intense signals from the gel matrix. Consequently, the FT-IR spectra of the DES gels prepared with different lignin exhibit identical characteristic peaks. No new peaks appear after lignin incorporation, nor are any obvious peak shift observed among the different samples. This indicates that the addition of a small amount of lignin does not significantly alter the main chemical structure of the gel system.

#### 2.2.4. Thermal Stability Analysis of DES Gels

**Figure 9:**
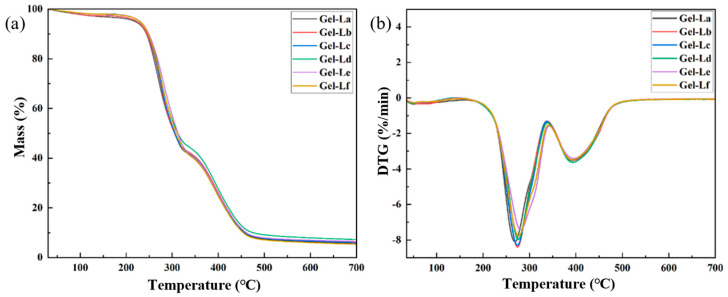
TG curves (**a**) and DTG curves (**b**) of DES gels with different kinds of lignin.

The thermogravimetric (TGA) and derivative thermogravimetric (DTG) profiles for DES gels with different lignin additions are depicted in Figure 9. The TGA and DTG curves for the DES gels exhibit the three typical stages of hydrogel thermal decomposition: preheating, rapid pyrolysis, and char formation [43,44]. The thermal decomposition processes and final char residue of the gels with different lignins are quite similar. The final char residue in the DES gels is approximately 7%, which may be due to the relatively low lignin content, resulting in no significant difference in thermal stability.

## 3. Conclusions

In this study, various DES systems were utilized for the processing of *Paulownia* wood powder, and the extracted lignin was analyzed to investigate its chemical structure. The study explored how different DES systems influenced lignin structure and examined the effects of lignin structure on the properties of DES gels. By analyzing the chemical structure of lignin extracted from *Paulownia* wood using various DES systems, it was found that the lignin retained a relatively complete aromatic ring structure, with similar structural characteristics across the systems. However, lignin extracted with acidic DES systems underwent significant cleavage of β-O-4′ linkages during pretreatment, leading to lignin precipitation. The molecular weight distribution of the lignins varied, with the ChCl-LA DES system cleaving β-O-4′ structures and concurrently promoting condensation reactions, thereby forming a large number of β-5′ linkages. In this process, both depolymerization and condensation reactions occurred, with the latter dominating, resulting in a higher lignin molecular weight. Compared to binary DES, the addition of acrylic acid in the ternary DES system caused lignin macromolecules to break down, resulting in lignin extracted from ternary DES systems having smaller molecular weights than those from binary DES systems. The thermal degradation trends of lignins extracted by different DES systems were similar, though their thermal stability varied, with the lignin extracted by the ChCl-LA system exhibiting the best thermal stability. When the lignin extracted from different DES systems was added to DES gels, the effects of lignin with different structures on the mechanical performance, swelling characteristics, microstructure, and thermal stability of the DES gels were assessed. It was found that the different structures of the *Paulownia* wood lignin had no significant impact on the properties of the DES gels. This result may be attributed to factors such as the dispersibility of lignin in the DES gel, the low concentration of lignin, and the interactions between lignin and DES molecules. This study provides valuable insights for the further development and utilization of lignin, as well as its application in the preparation of DES gels.

## 4. Materials and Methods

### 4.1. Experimental Materials

The experimental material was the trunk of a 6-year-old *Paulownia* 9501 (*Paulownia tomentosa* × *Paulownia fortune* 9501), harvested from the teaching and experimental base of Henan Agricultural University. After removing the bark, the *Paulownia* trunk was crushed and sieved to obtain wood powder with a particle size of 40–60 mesh. The powder was then continuously extracted for 6–8 h using a Soxhlet extractor with a 2:1 (*v*/*v*) toluene-ethanol mixture to eliminate other unbound or small-molecule lignin and waxes. After extraction, the sample was dried at 60 °C in an oven for further use.

The experimental reagents and instruments are shown in Table 3 and Table 4.

### 4.2. Experimental Methods

#### 4.2.1. Preparation of DES

Choline chloride (ChCl) was mixed with lactic acid (LA), glycerol (GL), urea, and acrylic acid (AA) in fixed molar ratios in a round-bottom flask. The blend was stirred continuously and heated maintained at 80 °C in a water bath until a clear, uniform solution formed, which was then sealed and stored in a desiccator for later use. The DES preparation ratios used in this experiment are shown in Table 5.

#### 4.2.2. DES Extraction of Lignin

Five grams of dried *Paulownia* wood powder were mixed with different DES systems at a solid-to-solvent mass ratio of 1:20 in a high-pressure reactor. The mixture was then heated and stirred on a magnetic stirrer at 140 °C for 2 h. After the reaction was complete, the reactor was placed in running water to cool down to room temperature. Then anhydrous ethanol was incorporated into the resultant mixture and thoroughly stirred. The mixture was filtered using a vacuum filtration device. The solid residue (mainly cellulose, with a small amount of hemicellulose and lignin) was desiccated at 80 °C in a hot-air oven. The filtrate was transferred to a rotary evaporator, where anhydrous ethanol was evaporated at 55 °C until completely removed. Subsequently, the evaporated solution was introduced into excess deionized water under continuous stirring for lignin precipitation. After the solution left to stand overnight, the precipitated lignin was centrifuged. Finally, the lignin was freeze-dried for 24 h and recovered. In this experiment, lignin extracted from different DES systems, including ChCl-LA, ChCl-GL, ChCl-Urea, ChCl-LA-AA, ChCl-GL-AA, and ChCl-Urea-AA, was named La, Lb, Lc, Ld, Le, and Lf, respectively, with L0 representing the original *Paulownia* material.

#### 4.2.3. Preparation of Lignin-DES Gel

Choline chloride was combined with acrylic acid at a molar ratio of 1:2 and heated in an 80 °C water bath with stirring until the mixture became a uniform and transparent liquid, forming the choline chloride-acrylic acid DES. For each of the six extracted lignins, 0.02 g of each lignin was dissolved in 20 g of DES. The lignin:DES ratio of 1:1000 (*w*/*w*) was selected based on preliminary experiments, in which this ratio gave the highest compressive strength, appropriate pore size, good lignin solubility, and a uniform gel network without aggregation. Subsequently, 0.12 g of the cross-linker MBA and 0.12 g of the initiator APS were added to 60 g of deionized water, and the mixture was mixed thoroughly. Next, the DES solutions containing different lignins were combined with the cross-linker and initiator solution, and blended uniformly, respectively. Ultimately, the resulting blends were subjected to an 80 °C constant-temperature water bath for 2 h to synthesize the DES gel samples. Based on the six lignin types added (La-Lf), the prepared DES gels were named Gel-La, Gel-Lb, Gel-Lc, Gel-Ld, Gel-Le, and Gel-Lf, respectively.

#### 4.2.4. Structural Characterization of Lignin

(1) Fourier Transform Infrared Spectroscopy (FT-IR) Detection

During the analysis, 1 mg of dried lignin was thoroughly ground with 100 mg of potassium bromide (KBr) powder to achieve a mass ratio of 1:100. The mixture was then compressed into pellets using a tablet press. FT-IR spectra of the compressed samples were recorded on a Nicolet iS10 Fourier-transform infrared spectrometer (Thermo Fisher Scientific, USA). The scanning range was set from 4000 to 400 cm^−1^, with a resolution of 4 cm^−1^ and 32 scans.

(2) Molecular Weight and Yield Determination

4 mg of dried lignin was dissolved in 2 mL of tetrahydrofuran (THF) and analyzed for molecular weight by gel permeation chromatography (GPC; Agilent 1200, USA). A PL-gel Mixed-D column (300 mm × 7.5 mm) was used, and the UV detector was set at a wavelength of 280 nm. Standard polystyrene solutions with four different molecular weights (1320, 6600, 9200, and 43,550 g/mol) were used for calibration. The lignin yield is calculated using the following formula:

$$\mathrm{Lignin}\;\mathrm{Yield}(\%)=\frac{m}{m_{0}\;\times \;C}\times \;100\%$$

where m represents the mass of lignin after freeze-drying (g), m_0_ represents the mass of *Paulownia* wood powder used in the pretreatment (g), and C represents the Klason lignin content in the *Paulownia* wood powder (determined to be 22.25%).

(3) Two-Dimensional Nuclear Magnetic Resonance (2D NMR) Detection

The NMR spectra were recorded on a Bruker AV III 400 MHz NMR spectrometer (Bruker Corporation, Germany) at 25 °C. 30 mg of the lignin sample was dissolved in 0.5 mL of deuterated dimethyl sulfoxide (DMSO-d_6_) and transferred into a standard 5 mm NMR tube. In the two-dimensional NMR spectra, the sweep widths for the ^1^H and ^13^C dimensions are 5000 Hz and 20,000 Hz, respectively. The number of sampling points in the ^1^H dimension is 1024, and the relaxation delay is 1.5 s, and the number of accumulated sampling points in the ^13^C dimension is 256, with a total of 64 scans. Zero-filling was applied to the data matrix before Fourier transformation. Spectral data processing was performed using the standard Bruker Topspin-NMR software (Topspin 2.1).

Quantitative information on the linkages and structural units in the lignin 2D NMR spectra was calculated using the following formulas:

$$I(C_{9})\;=\;0.5\;I(S_{2,6}\;+\;{S\prime }_{2,6})\;+\;I(G_{2})\;I(X)\%\;=\;[I(X)/I(C_{9})]\;\times \;100\%\;S/G\;=\;0.5I(S_{2,6}\;+\;{S\prime }_{2,6})/I(G_{2})$$

where I(S_2,6_ + S′_2,6_) represents the integral value of the signals from structural units S_2,6_ and S′_2,6_; I(G_2_) is the integral value of the G_2_ structural unit; I(C_9_) is the integral value of the C_9_ aromatic ring; I(X) represents the integral value of the α-carbon-hydrogen correlation signals of various linkages in lignin, such as β-O-4′, β-β′, and β-5′; and I(X)% represents the absolute quantitative value of each lignin linkage based on the C_9_ aromatic ring.

(4) Thermogravimetric Analysis (TGA)

Approximately 10 mg of lignin was analyzed using a synchronous thermal analyzer (TG-DSC STA8000, PerkinElmer, USA). The lignin sample was placed in a platinum pan, and the temperature was increased at 10 °C/min under a nitrogen atmosphere from 30 °C to 800 °C. The nitrogen flow rate was 20 mL/min, and the analysis was used to evaluate the thermal stability of the lignin.

#### 4.2.5. Characterization Methods of DES Gel

All measurements were conducted in triplicate, and the results are presented as averages.

(1) Mechanical Performance Testing

The mechanical characteristics of the DES gels were assessed utilizing an INSTRON 5982 universal testing machine (Instron, USA). The DES gel samples were cut into cylindrical shapes with a diameter of 30 mm and a height of 15 mm. The samples were compressed at a rate of 10 mm/min using a 1000 N capacity electronic universal testing machine to evaluate their compressive properties.

(2) Swelling Performance Testing

The DES gel samples were dried, and a predetermined weight of the desiccated gel was immersed in deionized water under ambient conditions. The gel sample was removed after the swelling equilibrium was reached, and the weight was recorded. The swelling performance of the gel was then analyzed.

(3) Microstructure Analysis

The DES gel samples were immersed in water until swelling equilibrium was reached, then dried in a vacuum freeze dryer for 24 h. Once desiccated, the gel samples underwent freeze-fracturing in liquid nitrogen, and the exposed cross-sections were subsequently sputtered with gold. The microstructure was examined using a ZEISS Sigma 300 scanning electron microscope (SEM, Carl Zeiss, Germany).

## Figures and Tables

**Table 1:** Molecular weights and lignin yield of different lignins.

	La	Lb	Lc	Ld	Le	Lf
Weight-average molecular weight Mw (g/mol)	1830	1810	1390	720	1000	840
Number-average molecular weight Mn (g/mol)	1060	700	900	540	550	510
Polydispersity index PDI	1.72	2.60	1.54	1.35	1.84	1.63
Lignin Yield (%)	75.57	7.3	3.36	45.55	7.64	6.83

**Table 2:** Quantitative analysis of linkages in different lignins by 2D NMR.

Sample Name	S/G	β-O-4′ ^a^	β-β′	β-5′
La	6.69	3.1	16.5	37.7
Lb	0.22	44.5	13.8	5.8
Lc	0.47	57.0	9.2	6.0
Ld	2.03	14.1	19.2	2.1
Le	0.61	11.7	15.8	6.1
Lf	0.61	50.1	10.4	4.5

^a^ Result expressed per 100 Ar based on quantitative 2D-HSQC spectra.

**Table 3:** Experimental reagents.

Name	Specifications	Manufacturer	Abbreviation
Choline chloride	Analytical grade	McLean Shanghai Biochemical Technology Co., Ltd. Shanghai, China.	ChCl
Lactic acid	Analytical grade	McLean Shanghai Biochemical Technology Co., Ltd. Shanghai, China.	LA
Glycerol	Analytical grade	Tianjin Damao Chemical Reagent Technology Co., Ltd., Tianjin, China.	GL
Urea	Analytical grade	McLean Shanghai Biochemical Technology Co., Ltd., Shanghai, China.	Urea
Acrylic acid	Analytical grade	McLean Shanghai Biochemical Technology Co., Ltd., Shanghai, China.	AA
Toluene	Analytical grade	Tianjin Damao Chemical Reagent Technology Co., Ltd., Tianjin, China.	MB
N,N′-Methylene-bisacrylamide	Analytical grade	McLean Shanghai Biochemical Technology Co., Ltd., Shanghai, China.	MBA
Ammonium persulfate	Analytical grade	McLean Shanghai Biochemical Technology Co., Ltd., Shanghai, China.	APS

**Table 4:** Experimental instruments.

Instrument Name	Model	Manufacturer
Electronic Analytical Balance	FA2004	Shanghai Shunyu Hengping Scientific Instruments Co., Ltd., Shanghai, China.
Electric Hot Air-Drying Oven	101-1AB	Tianjin Test Instrument Co., Ltd., Tianjin, China.
Vacuum Freeze Dryer	HXLG-10-50B	Shanghai Huxi Industrial Co., Ltd., Shanghai, China.
High-Speed Freezing Centrifuge	TGL-20	Sichuan Shuke Instrument Co., Ltd., Chengdu, China.
High-pressure reactor	200 ML	Changyi Laboratory Instruments Store Xi’an High-Tech Zone, Xi’an, China
Magnetic Stirrer	MS-H380-Pro	Beijing Daxing Longchuang Experimental Instrument Co., Ltd., Beijing, China.

**Table 5:** Composition of DES types.

DES Type	DES Composition	Molar Ratio of Components
Acidic	ChCl-LA	1:10
Neutral	ChCl-GL	1:2
Alkaline	ChCl-Urea	1:2
——	ChCl-LA-AA	2:1:1
——	ChCl-GL-AA	2:1:1
——	ChCl-Urea-AA	2:1:1

## Data Availability

All data and materials are available on request from the corresponding authors. The data are not publicly available due to ongoing research using a part of the data.

## References

[1] Ragauskas A.J., Beckham G.T., Biddy M.J., Chandra R., Chen F., Davis M.F., Davison B.H., Dixon R.A., Gilna P., Keller M. (2014). Lignin Valorization: Improving Lignin Processing in the Biorefinery. Science.

[2] Morena A.G., Tzanov T. (2022). Antibacterial Lignin-Based Nanoparticles and Their Use in Composite Materials. Nanoscale Adv..

[3] Jakubowski M. (2022). Cultivation Potential and Uses of Paulownia Wood: A Review. Forests.

[4] Rodríguez-Rebelo F., Rodríguez-Martínez B., Del-Río P.G., Collins M.N., Garrote G., Gullón B. (2024). Assessment of Deep Eutectic Solvents (DES) to Fractionate Paulownia Wood within A Biorefinery Scheme: Cellulosic Bioethanol Production and Lignin Isolation. Ind. Crops Prod..

[5] Novia N., Jannah A.M., Melwita E., Fudholi A., Pareek V.K. (2026). Advances and Challenges in Deep Eutectic Solvents Pretreatment Technologies for Bioethanol Production from Lignocellulosic Biomass: A Comprehensive Review. Renew. Sustain. Energy Rev..

[6] Abbott A.P., Boothby D., Capper G., Davies D.L., Rasheed R.K. (2004). Deep Eutectic Solvents Formed between Choline Chloride and Carboxylic Acids: Versatile Alternatives to Ionic Liquids. J. Am. Chem. Soc..

[7] Smith E.L., Abbott A.P., Ryder K.S. (2014). Deep Eutectic Solvents (DESs) and Their Applications. Chem. Rev..

[8] Wang S., Li H., Xiao L.-P., Song G. (2020). Unraveling the Structural Transformation of Wood Lignin During Deep Eutectic Solvent Treatment. Front. Energy Res..

[9] Zhang X., Liu A., Li X., Xu W., Duan X., Shi J., Li X. (2025). Research Progress of Deep Eutectic Solvents in Lignocellulosic Biomass Pretreatment. Cellulose.

[10] Alvarez-Vasco C., Ma R., Quintero M., Guo M., Geleynse S., Ramasamy K.K., Wolcott M., Zhang X. (2016). Unique Low-Molecular-Weight Lignin with High Purity Extracted from Wood by Deep Eutectic Solvents (DES): A Source of Lignin for Valorization. Green Chem..

[11] Xu H., Li B., Mu X. (2016). Review of Alkali-Based Pretreatment To Enhance Enzymatic Saccharification for Lignocellulosic Biomass Conversion. Ind. Eng. Chem. Res..

[12] Li H., Zhou C., Wang L., Yang F., Liang J., Wang F., Li P., Li C., Wu Z., Ren T. (2024). A Novel Eco-Friendly Bamboo-Based Composite Biochar for Effective Removing Oxytetracycline Hydrochloride. Adv. Compos. Hybrid Mater..

[13] Huang J., Liu W., Qiu X. (2019). High Performance Thermoplastic Elastomers with Biomass Lignin as Plastic Phase. ACS Sustain. Chem. Eng..

[14] Li C., Li M., Li Z., Guo P., Zhao Z., Lu W., Li J., Liang J., Tang Y., Ge S. (2024). Cleaner Production of Liquefied Biomass-Based Phenol–Formaldehyde Resin with Improved Properties via Catalyzed Copolymerization. Adv. Compos. Hybrid Mater..

[15] Xiao G., Xie S., Mao B., Chen H., Xue Y., Xu Q., Guo J., Dai M. (2025). Tailoring Functionalized Lignin-Based Spherical Resins as Recyclable Adsorbents for Heavy Metal Uptake. Polymers.

[16] Liu C., Ni S., Wang Z., Fu Y., Qin M., Zhang Y. (2025). Direct In Situ Conversion of Both Lignin and Hemicellulose into Single Functional Biopolymers via Biomass Fractionation Process. Polymers.

[17] Ingtipi K., Moholkar V.S. (2019). Sonochemically Synthesized Lignin Nanoparticles and its Application in the Development of Nanocomposite Hydrogel. Mater. Today Proc..

[18] Li C., Zhang X., Zhou C., Yang F., Liang J., Gu H., Wang J., Wang F., Peng W., Guo J. (2024). Performance and Mechanism of a Novel Bamboo-Based Magnetic Biochar Composite for Efficient Removal of Norfloxacin. Adv. Compos. Hybrid Mater..

[19] Li X., Luo N., Li Z., Li P., Chang J., Fang L., Huang Q., Zhu B., Zhang Y., Zhou X. (2026). Polymerizable Deep Eutectic Solvents-Enabled High-Lignin-Density Networks for Ambient Multi-Scale Fabrication of Multifunctional and Extreme Environment Adaptable Soft Devices. Adv. Mater..

[20] Yan Y., He C., Zhang L., Dong H., Zhang X. (2023). Freeze-Resistant, Rapidly Polymerizable, Ionic Conductive Hydrogel Induced by Deep Eutectic Solvent (DES) after Lignocellulose Pretreatment for Flexible Sensors. Int. J. Biol. Macromol..

[21] Wang H., Li J., Yu X., Yan G., Tang X., Sun Y., Zeng X., Lin L. (2021). Cellulose Nanocrystalline Hydrogel Based on A Choline Chloride Deep Eutectic Solvent as Wearable Strain Sensor for Human Motion. Carbohydr. Polym..

[22] Huang J., Hu Y., Li J., Wang H., Wang T., Wu H., Li Y., Wang M., Zhang J. (2023). A Flexible Supercapacitor with High Energy Density Driven by MXene/Deep Eutectic Solvent Gel Polyelectrolyte. ACS Energy Lett..

[23] Wang J., Deng Y., Ma Z., Wang Y., Zhang S., Yan L. (2021). Lignin Promoted the Fast Formation of A Robust and Highly Conductive Deep Eutectic Solvent Ionic Gel at Room Temperature for A Flexible Quasi-Solid-State Supercapacitor and Strain Sensors. Green Chem..

[24] Yang L., Xing M., Xue X., Jin X., Wang Y., Xiao F., Li C., Wang F. (2025). Preparation and Characterization of a Novel Eco-Friendly Acorn-Based Wood Adhesive with High Performance. Forests.

[25] Van Erven G., Boerkamp V.J., Van Groenestijn J.W., Gosselink R.J. (2024). Choline and Lactic Acid Covalently Incorporate into the Lignin Structure during Deep Eutectic Solvent Pulping. Green Chem..

[26] Wu Y., Song R., Tai Y., Wang W., Zhong L. (2025). Separation of High-Yield and High-Purity Lignin from Elm Wood Using Ternary Deep Eutectic Solvents. Nord. Pulp Pap. Res. J..

[27] Li C., Li Z., Wang L., Xue X., Xiao F., Guo J., Li J., Dong Y., Li J., Bao C. (2026). High-Performance Phenolic Resin Reinforced by Tannic Acid-Polyethyleneimine Functionalized Multi-Walled Carbon Nanotubes for Wood-Based Panels. Macromol. Rapid Commun..

[28] Zhang C., Guo K.-N., Ma C.-Y., Bian J., Wen J.-L., Yuan T.-Q. (2023). Assessing the Availability of Bamboo (Phyllostachys Pubescens) Fibers and Parenchyma Cells for Producing Lignin Nanoparticles and Fermentable Sugars by Rapid Carboxylic Acid-Based Deep Eutectic Solvents Pretreatment. Ind. Crops Prod..

[29] Pan X., Kadla J.F., Ehara K., Gilkes N., Saddler J.N. (2006). Organosolv Ethanol Lignin from Hybrid Poplar as a Radical Scavenger: Relationship between Lignin Structure, Extraction Conditions, and Antioxidant Activity. J. Agric. Food Chem..

[30] da Costa Lopes A.M., Gomes J.R., Coutinho J.A., Silvestre A.J. (2020). Novel Insights into Biomass Delignification with Acidic Deep Eutectic Solvents: A Mechanistic Study of β-O-4 Ether Bond Cleavage and the Role of the Halide Counterion in the Catalytic Performance. Green Chem..

[31] Jančíková V., Jablonský M. (2024). Exploiting Deep Eutectic Solvent-Like Mixtures for Fractionation Biomass, and the Mechanism Removal of Lignin: A Review. Sustainability.

[32] Guo J., Yu G., Wang J. (2025). Comparative Insight into Biomass Pretreatment by Choline Chloride-Based Deep Eutectic Solvents in Relation to Their Physicochemical Characteristics. J. Environ. Chem. Eng..

[33] Rencoret J., Marques G., Gutiérrez A., Nieto L., Jiménez-Barbero J., Martínez Á.T. (2009). Isolation and Structural Characterization of the Milled-Wood Lignin from Paulownia Fortunei Wood. Ind. Crops Prod..

[34] Ma C.-Y., Gao X., Peng X.-P., Gao Y.-F., Liu J., Wen J.-L., Yuan T.-Q. (2021). Microwave-assisted deep eutectic solvents (DES) pretreatment of control and transgenic poplars for boosting the lignin valorization and cellulose bioconversion. Ind. Crops Prod..

[35] Ouensanga A., Picard C. (1988). Thermal degradation of sugar cane bagasse. Thermochim. Acta.

[36] Wang S., Dai G., Yang H., Luo Z. (2017). Lignocellulosic Biomass Pyrolysis Mechanism: A State-of-the-Art Review. Prog. Energy Combust. Sci..

[37] Lei Z., Shao J., Li C., Jiang S., Yao M., Li J. (2025). Skin-Inspired Durable and Cost-Effective Biomass-Based Supramolecular Adhesives. Adv. Funct. Mater..

[38] Li X., Yan M., Wu X., Pan M., Mota-Morales J.D., Lian H. (2023). Construction and Application of Biobased PDES Ionic Gels with a Soft–Hard Segment. ACS Appl. Polym. Mater..

[39] Wang R., Cheng C., Wang H., Wang D. (2024). Swollen hydrogel nanotechnology: Advanced applications of the rudimentary swelling properties of hydrogels. ChemPhysMater.

[40] Aziz T., Farid A., Haq F., Kiran M., Ullah A., Zhang K., Li C., Ghazanfar S., Sun H., Ullah R. (2022). A Review on the Modification of Cellulose and Its Applications. Polymers.

[41] Luo X., Liu C., Yuan J., Zhu X., Liu S. (2017). Interfacial Solid-Phase Chemical Modification with Mannich Reaction and Fe(III) Chelation for Designing Lignin-Based Spherical Nanoparticle Adsorbents for Highly Efficient Removal of Low Concentration Phosphate from Water. ACS Sustain. Chem. Eng..

[42] Yu S., Qiu B., Jin Y., Zhao Y., Luo W., Qi X. (2025). Efficient Removal of Lignin in Tobacco Stems with Choline Chloride-Based Deep Eutectic Solvents. Ind. Crops Prod..

[43] Caulfield M.J., Qiao G.G., Solomon D.H. (2002). Some Aspects of the Properties and Degradation of Polyacrylamides. Chem. Rev..

[44] Bi Q., Luo X., Yu J., Qin Z., Li C., Mo L. (2025). Construction of Zno@Pda Core-Shell Nanoparticle in Antimicrobial Nanofibril Aerogel for Sustainable Oil-Water Separation. Ind. Crops Prod..

